# Computer-assisted photoreceptor assessment on Heidelberg Engineering Spectralis™ High Magnification Module™ images

**DOI:** 10.1007/s00417-021-05326-6

**Published:** 2021-08-06

**Authors:** Timo W. F. Mulders, B. Jeroen Klevering, Carel B. Hoyng, Thomas Theelen

**Affiliations:** Department of Ophthalmology, Donders Institute for Brain, Cognition and Behaviour, Radboud University Medical Centre (Radboudumc), Philips van Leydenlaan 15, 6525 EX Nijmegen, The Netherlands

**Keywords:** High Magnification Module™, Cone metrics, Computer-assisted photoreceptor analysis, Repeatability

## Abstract

**Purpose:**

To evaluate reliability and repeatability of computer-assisted measurements of cone photoreceptor metrics on Heidelberg Engineering Spectralis™ High Magnification Module (HMM™) Automatic Real-time Tracking (ART™) images.

**Methods:**

We analyzed HMM™ images in three separate study arms. Computer-assisted cone identification software was validated using an open-access adaptive optics (AO) dataset. We compared results of the first arm to data from AO and histology. We evaluated intersession repeatability of our computer-assisted cone analysis in the second arm. We assessed the capability of HMM™ to visualize cones in the presence of pathology in the third arm.

**Results:**

We included 10 healthy subjects in the first arm of our study, 5 additional healthy participants in the second arm and 5 patients in the third arm. In total, we analyzed 225 regions of interest on HMM™ images. We were able to automatically identify cone photoreceptors and assess corresponding metrics at all eccentricities between 2 and 9° from the fovea. Cone density significantly declined with increasing eccentricity (*p* = 4.890E-26, Friedman test). With increasing eccentricity, we found a significant increase in intercell distance (*p* = 2.196E-25, Friedman test) and nearest neighbor distance (*p* = 1.997E-25, Friedman test). Cone hexagonality ranged between 71 and 85%. We found excellent automated intersession repeatability of cone density counts and spacing measurements. In pathology, we were also able to repeatedly visualize photoreceptors.

**Conclusion:**

Computer-assisted cone photoreceptor analysis on Spectralis™ HMM™ images is feasible, and most cone metrics show excellent repeatability. HMM™ imaging may be useful for photoreceptor analysis as progression marker in outer retinal disease.


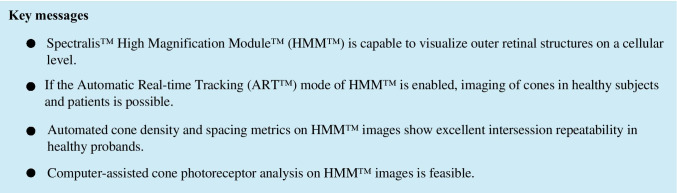


## Introduction

In vivo imaging of single photoreceptors of the human retina has been made possible by ocular imaging modalities equipped with adaptive optics (AO) technique [1]. AO overcomes multiple limitations of optical irregularities of the human eye, resulting in a high lateral resolution of the retinal image [2, 3]. However, AO is technically challenging and is therefore expensive and not broadly available [4]. These limitations make the use of AO in multi-center clinical studies difficult even though, it would be of benefit to approach the loss of individual retinal photoreceptors and accompanying changes of photoreceptor mosaic as biomarkers of early retinal disorder or as treatment response of retinal disease [5].

Confocal scanning laser ophthalmoscopy (cSLO) is a widespread fundus imaging technique with a relatively high lateral resolution of about 5 µ and an axial resolution of about 300 µ [1, 4]. Via a confocal pinhole, cSLO blocks out-of-focus light in the image formation and thus enables imaging of the focal plane only. In most cases, cSLO is not able to image individual cone photoreceptors when equipped with standard lenses. Recently, LaRocca et al. were able to visualize single human retinal cones in vivo by a custom-made cSLO with a narrower field-of-view (FOV), smaller confocal pinhole, and increased scanning velocity compared to the commercially available cSLO Spectralis™ (Heidelberg Engineering, Heidelberg, Germany) [6].

Recently, the Spectralis™ High Magnification Module (HMM™, Heidelberg Engineering, Heidelberg, Germany) was introduced. This module consists of a software tool for the Heidelberg Eye Explorer™ and a lens with increased magnification that replaces the standard 30° lens of the Spectralis™ device. Images acquired by HMM™ cover a fundus area of 8 × 8°, allowing for a digital lateral resolution of 1.5 μm/px if the high-resolution mode of the device is chosen for imaging [7]. While HMM™ was capable to visualize outer retinal structures on a cellular level, it remained doubtful if HMM™ images could provide reliable and repeatable quantitative parameters of retinal photoreceptors [8–10].

As a proof of principle, we evaluate whether computer-assisted cone measurements on HMM™ images, based on automated and validated cell counts, were reliable and repeatable and could thus be of sufficient value in future clinical trials. Furthermore, we assessed the capability of HMM™ to visualize cones in the presence of retinal pathology.

## Methods

### Participants

We recruited subjects among employees and patients from the Department of Ophthalmology, Radboud University Medical Centre (Radboudumc). We obtained ethical approval by the local institutional review board (project number: 2017–3535) and conducted this study according to the tenets of the Declaration of Helsinki. All participants received and accepted a written informed consent before inclusion in the study.

### Inclusion and exclusion criteria

We performed all study measurements on the right eye of all participants. We defined three study groups: we used measurement results of group 1 to establish initial reference values to enable a comparison to the results of AO and histological studies. Group 2 served to assess intersession repeatability of the measurements. We included patients with macular pathology in group 3 to see if it was possible to identify and analyze photoreceptors on HMM™ images in ART™ mode in a retina affected by disease. We performed objective refraction measurement (Nidek Tonoref II, NIDEK CO., LTD. Gamagori, Japan), best-corrected visual acuity (BCVA), and macular 30° infrared reflectance cSLO imaging and spectral domain optical coherence tomography (SD-OCT) by the Spectralis™ device (Heidelberg Engineering GmbH, Heidelberg, Germany). We excluded any subjects in groups 1 and 2 with obvious funduscopic abnormalities or a BCVA worse than 6/6 Snellen visual acuity. SD-OCT in group 3 was used to assess structural integrity of the photoreceptor layer.

### High-magnification scanning laser ophthalmoscope and imaging protocol

We used the commercial Spectralis™ HMM™ to capture 8 × 8° fundus images at standardized gaze positions, i.e., primary central gaze, as well as inferior, superior, temporal, and nasal gaze by using the preinstalled positions of the internal fixation lights of Spectralis™. We performed imaging with undilated pupils and placed a desk lamp before the not-examined eye to reduce the pupil diameter in the examined eye to less than 3 mm, to minimize the impact of peripheral optical aberrations. We corrected spherical aberrations of study eyes by adjustment of the focusing knob of the Spectralis™ device. Before imaging, we entered corneal diameter measurements acquired from Nidek Tonoref of each participant in the Heyex™ software, to ensure reliable distance measurements. We used the equivalent spherical aberration of study eyes as a baseline setting for focusing on cone photoreceptors, and only minimal focus corrections of utmost ± 0.5 D were applied to acquire optimal focusing in the center of the image. We repeatably adjusted the position of the Spectralis™ device in correspondence to ocular gaze direction when capturing eccentric images so that scanning direction was always parallel to the retinal surface in the center of the image to enhance focus on the photoreceptor layer in the arcuate neurosensory retina.

To further enhance image quality, we averaged 5 registered, unprocessed HMM™ images per gaze direction, using the ART™ function. We manually selected HMM™ ART™ images to be computed as compositions by the Heyex™ composition tool. We then uploaded the images to Fiji software (version 15.1n, National Institutes of Health, Bethesda, MD, USA) [11]. We first adapted the pixel scale to match the original scale in Heyex™ and then stitched a single composition image of the various gaze directions via the “2D stitching plugin” [12]. We used the OCT volume scan of the same area to identify the foveal center, and we aligned the marked location of the fovea on OCT with the HMM composition via the “Big Warp” plugin, using blood vessels as reference points [13]. We made use of the “Concentric Circles” plugin to define perifoveal circles with radii from 1 to 9° eccentricity, in steps of 1° [11]. To convert distance from the fovea in micrometers to degrees of eccentricity, we used an angular scale of 291 μm per degree of visual angle [14]. We selected regions of interest (ROI) of 240 × 240 μm at superior, inferior, temporal, and nasal directions at ~ 2, 3, 5, 7, and 9° eccentricity, avoiding blood vessels and imaging artifacts. For analysis purposes, we converted ROIs to 16-bit sharpened images.

We assessed intersession repeatability with two separate HMM™ imaging sessions of the same areas, conducted by the same operator within a timeframe of 7 days. We acquired central 8 × 8° images of the subjects and computed a single composition image at baseline and follow-up, according to the previously described methodology. We used the same protocol as described for group 1 to manually identify and align the fovea on OCT with HMM™ imaging. Following alignment, we projected scaled concentric circles at 2 and 3° on central HMM™ images and selected ROIs of 240 μm by 240 μm in every participant at both 2 and 3° on baseline and follow-up images.. Intraclass correlation coefficients of cone metrics in high-resolution ART™ mode were established.

### Photoreceptor-based metrics

We explored photoreceptor-based metrics in HMM™ imaging and compared the results to earlier publications on AO in vivo imaging and on histological data of human retinas. We used the 3D maxima finder plugin to identify photoreceptors as local maxima in a specified radius of 2.63, 3.17, 3.55, 3.68, and 3.74 μm at 2, 3, 5, 7, and 9° eccentricity, respectively [15]. The size of the measurement radii we used was based on previously published cone inner segment diameter measurements [16]. This technique aims to reduce the number of false positives by small, noise-induced maxima but also reduces the number of false negatives by large signals that are very close to each other. To further increase the reliability of measurements, we excluded any maxima on the ROI edges from analysis. We conducted all measurements using a noise value threshold of 1.00.

**Fig. 1:**
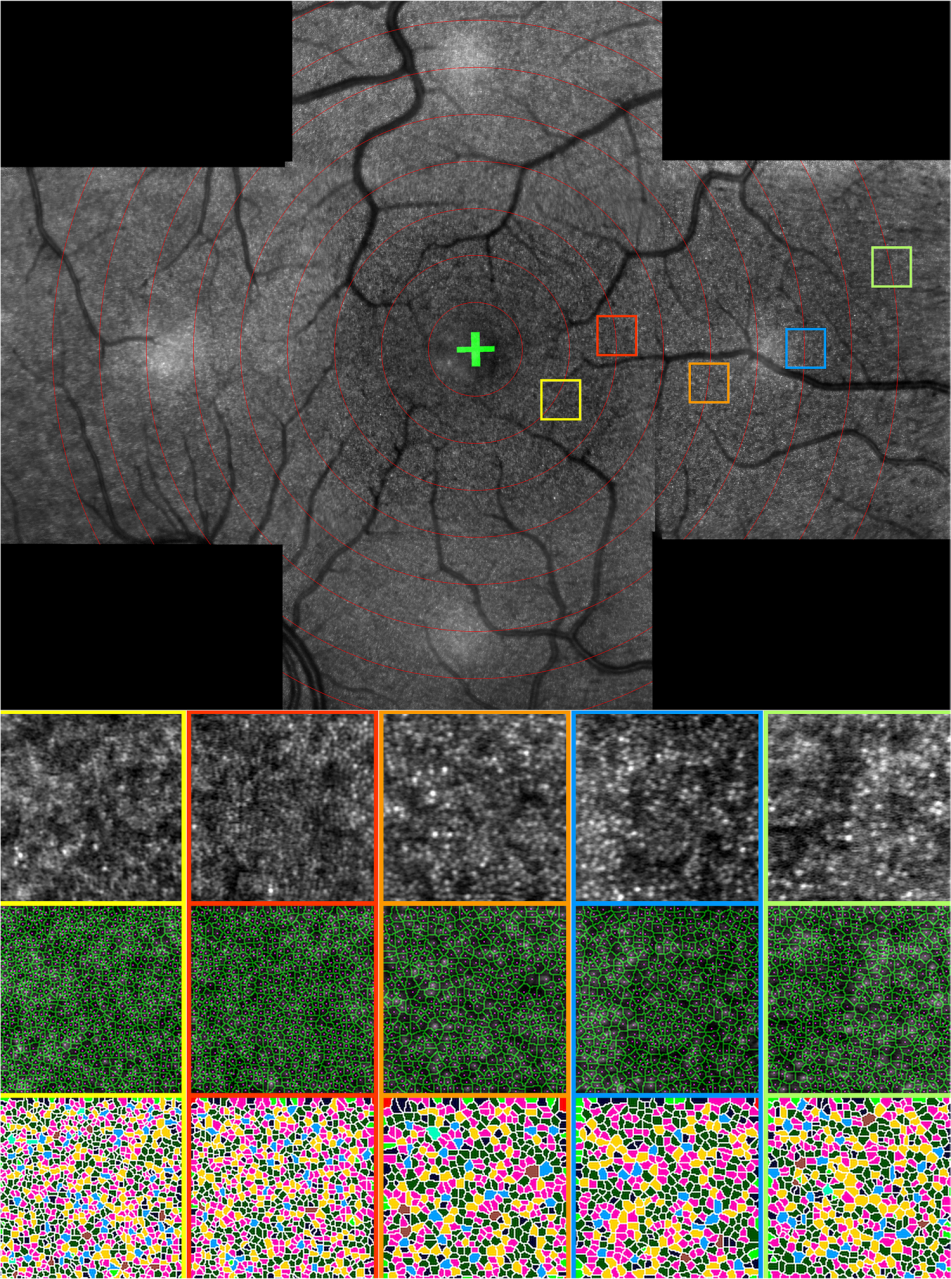
Illustration of cone analysis from HMM™ images at different eccentricities. (First row) HMM™ composite image. ROIs for cone measurements (colored squares) are located on defined eccentricities (red circles) from the fovea (green cross). (Second row) Enlarged photoreceptor images within ROIs at 2 (yellow), 3 (orange), 5 (ochre), 7 (light blue), and 9° (green) nasal eccentricity. (Third row) Computer-assisted cone photoreceptor identification. Cones recognized by the software are marked by purple dots. The borders of the corresponding Voronoi cells are marked in green. (Fourth row) Number of neighbor (NON) analysis depicted in color code. NON 5 = pink; NON 6 = dark green; NON 7 = yellow

Image analysis was performed as shown in Fig. 1. We evaluated cone density, packing, and spacing measurements as potential HMM™ photoreceptor-based metrics [5, 17]. We calculated cone density (cones/mm^2^) as the ratio of the number of maxima divided by ROI size in square millimeters. We defined packing as number of neighboring cells resulting from Voronoi analysis. Using “Biovoxxel neighbor analysis,” we calculated the percentage of cones showing hexagonal tiling (defined as *n* = 6 ± 1) [18]. We used intercell distance (ICD) and nearest neighbor distance (NND) to evaluate cone spacing. Via “Delaunay triangulation” and “BioVoxxel 2D particle distribution analysis” plugins, we approximated ICD and NND, respectively [11, 18].

### Validation

For external validation of our automated cone count methodology, we made use of 760 images from an open access AO dataset provided by Cunefare et al., available at https://github.com/DavidCunefare/CNN-Cone-Detection, accessed on April 14, 2020 [19]. In this dataset, cone density counts were conducted after an expert grader semi-automatically marked cone photoreceptors in all images [20]. Differences between automated cone density by our approach and provided manual cone density counts were compared via Bland–Altman analysis.

### Statistical analysis

We performed statistical analysis using SPSS version 25 (IBM, Armonk, NY, USA). We considered an unadjusted *P* value of < 0.05 statistically significant. To assess normal distribution, we used Shapiro–Wilk normality test. We used Friedman test to evaluate overall differences between ROI characteristics, followed by post hoc testing of consecutive eccentricities via Wilcoxon signed rank test. In order to minimalize type 1 errors, we applied Holm-Bonferroni correction for multiple testing. We calculated ICC estimate via a single-measurements, absolute-agreement, 2-way random-effects model.

## Results

**Fig. 2:**
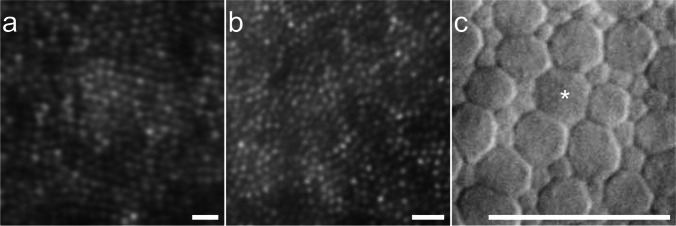
Parafoveal cone photoreceptor mosaic imaging by HMM™ compared to adaptive optics and histology. **A** Spectralis™ HMM™ ART™ imaging at 3°. **B** Flood-illumination AO-SLO imaging at 1° (adapted from Carrol et al. 2009), C. Differential interference contrast microscopy image of cones (asterisk) at ~ 2° eccentricity (adapted from Curcio et al. 1990) [21, 22]. Scale bar: 20 μm

We were able to identify cone photoreceptors as multiple, tiny, dots of inconsistently increased reflectivity, organized in a mosaic-like pattern, similar to the two-dimensional pattern of human retinal cone photoreceptors on AO and histology (Fig. 2). Contrary to AO and histology, cones located near the foveal center unfortunately could not be resolved by Spectralis™ HMM™. We computed compositions of 3 to 40 ART™ HMM™ images for each gaze direction. Approximately 15 min were necessary to acquire HMM™ images in 5 gaze directions. Post hoc analysis, which included stitching a single composition image of the various gaze directions, identifying the fovea, selecting ROIs, and analyzing cone metrics, required approximately an additional 15 min per eye.

### Validation

**Fig. 3:**
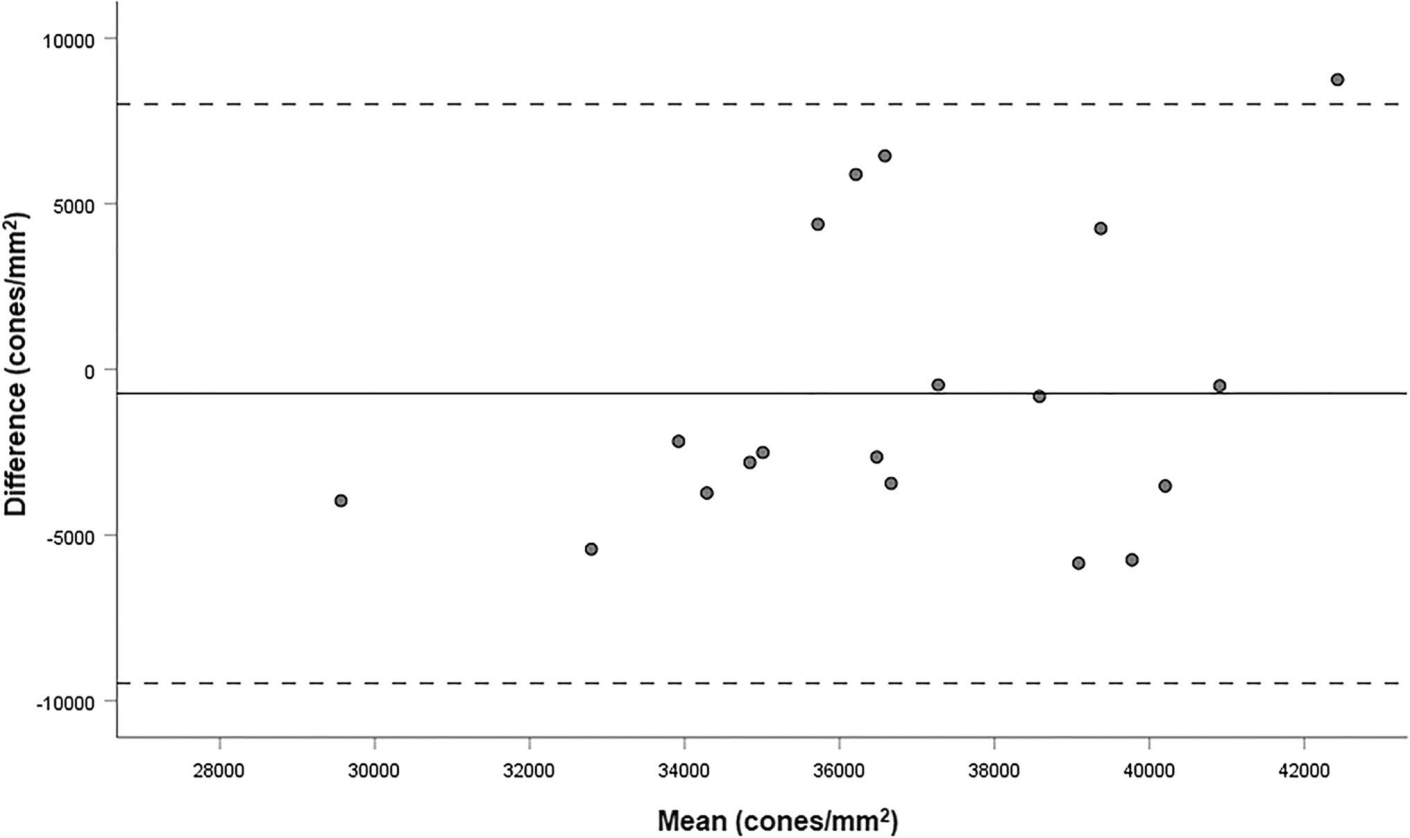
Bland–Altman plot of manual versus automatic cone density counts. The mean difference is shown by the bold, central line. The dotted lines represent the upper and lower 95% CI

Mean difference in cone density count between manual counts from Garrioch et al. and our method was − 730.29 (95% CI: − 9460.97–8000.40) cones/mm^2^ and did not reached statistical significance (*p* = 0.484, one-sample *t*-test) [20]. We found no statistically significant correlation between the difference and mean of both counts using linear regression analysis (*p* = 0.171). We therefore concluded that there was no proportional bias present. The results are depicted as a Bland–Altman plot in Fig. 3.

### Photoreceptor-based metrics

We included ten right eyes of ten healthy participants (6 males and 4 females; median age 28 (range: 26–35) years) in group 1, who underwent HMM™ imaging of their right eye. No eyes were excluded from our study. The median spherical equivalent refractive error was − 0.06 (range, − 2.00– + 0.38) D. An overview of cone metric analysis is presented in Fig. 1. Data on cone density, packing, and spacing metrics of 200 ROIs are presented in Table 1.

**Table 1:** Cone metrics on HMM™ images

Cone metric	Eccentricity *Median* + *range*					ICC *Mean* + *95% CI*
	2°	3°	5°	7°	9°	
Density (cones/mm^2^)	20,022 (13,431–30,136)	14,946 (10,741–22,784)	11,452 (8448–14,501)	10,818 (7690–13,427)	10,790 (7914–16,586)	0.965 (0.872–0.991)
Hexagonality (%)	77 (74–83)	77 (74–84)	77 (73–82)	79 (75–85)	78 (71–84)	0.545 (0.00–0.864)
ICD (μm)	8.31 (6.76–10.20)	9.70 (7.74–11.47)	11.03 (9.80–13.03)	11.36 (10.24–13.69)	11.46 (9.10–13.24)	0.953 (0.825–0.988)
NND (μm)	4.58 (3.87–5.71)	5.56 (4.41–6.68)	6.57 (5.63–7.81)	6.72 (5.54–8.14)	6.68 (5.30–8.51)	0.914 (0.697–0.978)

Abbreviations: *ICC*, intraclass correlation coefficient; *CI*, confidence interval; *ICD*, intercell distance; *NND*, nearest neighbor distance

### Cone density

Overall, we found a significant difference in cone photoreceptor density with increasing eccentricity (*p* = 4.890E-26, Friedman test). Post hoc analysis revealed a significant decline between 2, 3, and 5° (*p* = 3.570E-8 and *p* = 3.569E-8, Wilcoxon signed rank test). Results of automated cone density at 2, 3, 5, 7, and 9° eccentricity are presented in Table 1.

### Cone packing

Voronoi analysis regarding cone hexagonality (*n* = 6 ± 1) resulted in an overall hexagonality range between 2 and 9° of 71 − 85% (Table 1). We found a statistically significant difference in hexagonality between 5 and 7° (*p* = 7.000E-5, Wilcoxon signed rank test).

### Cone spacing

We found a significant difference in ICD when eccentricity increased (*p* = 2.196E-25, Friedman test). ICD measurements between 2, 3, and 5° reached statistical significance (*p* = 3.569E-8 between 2 and 3° and *p* = 8.182E-8 between 3 and 5°, Wilcoxon signed rank test). Using the nearest neighbor analysis, we revealed a statistically significant difference (*p* = 1.997E-25, Friedman test) in cone spacing when eccentricity increased. Similar to ICD, we found a statistically significant decrease in NND between 2 to 3° and 3 to 5° (*p* = 3.558E-8 and *p* = 5.214E-8, Wilcoxon signed rank test). We identified a NND range from 5.30 to 8.14 μm between 5 and 9°.

### Repeatability

We included five additional healthy participants (3 males and 2 females; median age 31 (range: 25–53) years) in group 2. We compared 10 ROIs at 2 and 3° from in the baseline imaging session, to 10 corresponding ROIs acquired in the follow-up imaging session. The results of the intersession repeatability are presented in Table 1. Median spherical equivalent refractive error was − 0.25 (range: − 0.125– − 4.875) D. Cone density counts and spacing measurements showed excellent repeatability, with a corresponding ICC greater than 0.9. Absolute differences between baseline and follow-up cone density, hexagonality, ICD, and NND did not reached statistical significance via a one-sample *t*-test (*p* = 0.398, *p* = 0.484, *p* = 0.780, and *p* = 0.691, respectively).

### Pathology

**Fig. 4:**
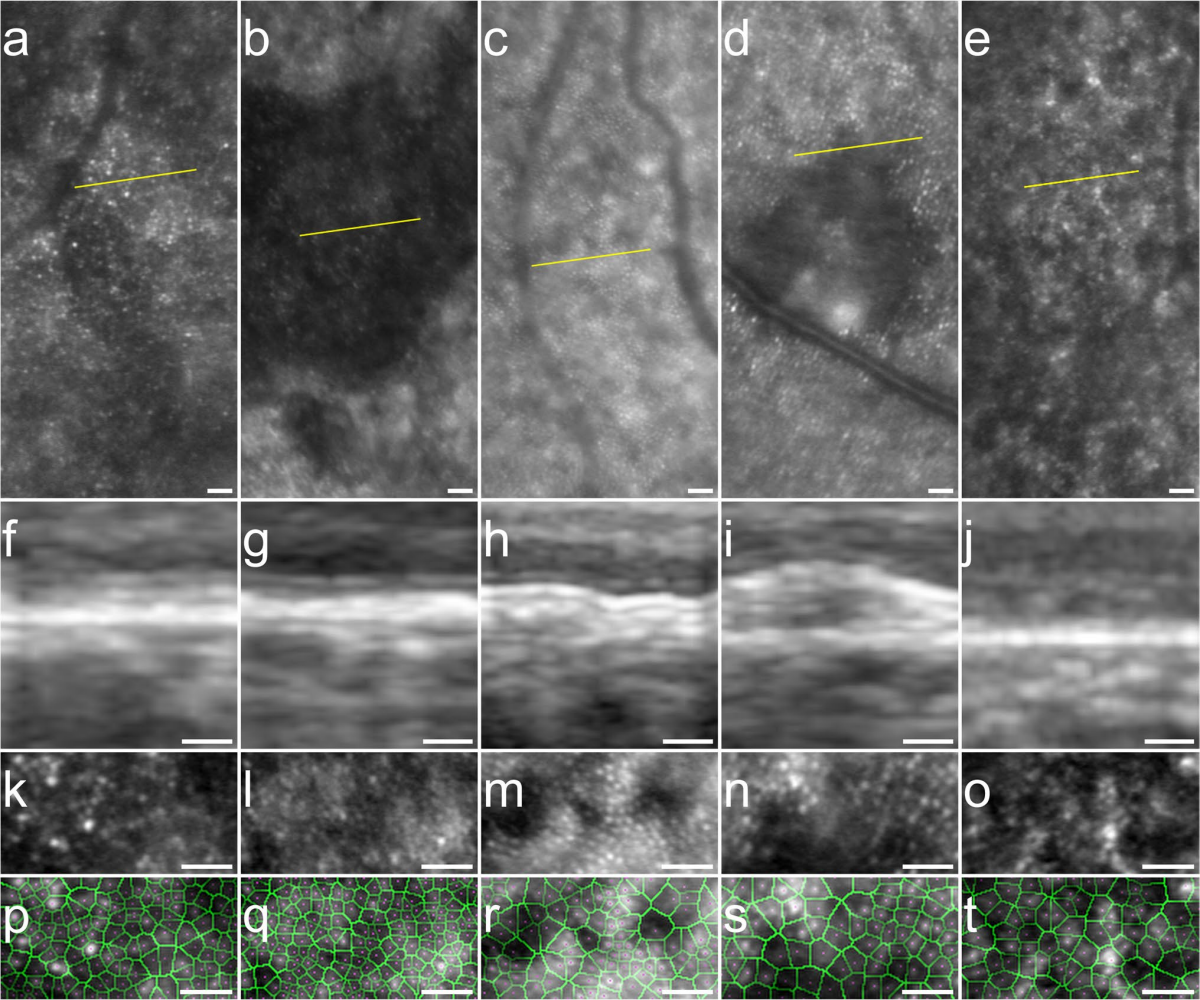
HMM™ and OCT imaging of cone photoreceptors in retinal pathology. HMM™ images (**A**–**E**) with a yellow line indicating locations of corresponding spectral domain OCT scans (**F**–**J**). Photoreceptor mosaic on HMM™ images, aligned with OCT scans (**K**–**O**) with results of computer-assisted cone evaluation of corresponding retinal areas (**P**–**T**). Cones recognized by the software are marked by purple dots. The borders of the corresponding Voronoi cells are marked in green. Central areolar choroidal dystrophy (CACD); BCVA OD: 20/15; images acquired at ~ 2° (**A**, **F**, **K**, **P**). CACD; BCVA OD: 20/40; images acquired at ~ 2° (**B**, **G**, **L**, **Q**). Early-onset AMD; BCVA OD: 20/25; images acquired at ~ 9° (**C**, **H**, **M**, **R**). Age-related macular degeneration (AMD); BCVA OD: 20/32; images acquired at ~ 5° (**D**, **I**, **N**, **S**). Stargardt disease; BCVA OD: 20/63; images acquired at ~ 5° (**E**, **J**, **O**, **T**). Scale bar: 50 μm

We captured HMM™ ART™ images of 5 right eyes of five patients suffering from central areolar choroidal dystrophy (CACD), age-related macular degeneration (AMD), and Stargardt disease. Median age of group 3 was 53 (range: 43–57) years. Images were acquired and analyzed using the same imaging protocols as previously described. Results of the analysis of 5 ROIs are presented in Fig. 4. We could observe photoreceptor cells in every patient and abnormalities in the photoreceptor layer as seen on OCT corresponded with apparent disruptions in the photoreceptor mosaic on HMM™ images. In areas with photoreceptor abnormalities on OCT, computer-assisted analysis revealed an cone density range of 2461–5901 cones/mm^2^, a hexagonality range of 58–71%, an ICD range of 16.07–26.00 μm, and a NND range of 8.87–14.71 μm on HMM™ images. Despite the clear visibility of photoreceptor cells, our algorithm failed to accurately identify several photoreceptors in the presence of pathology, as seen in panel P–T in Fig. 4.

## Discussion

In our current study, we investigated the reliability and repeatability of Spectralis™ HMM™ based cone photoreceptors mapping in the posterior pole via ART™ settings as a proof-of-concept. We were able to perform computer-assisted analysis of readily established cone mosaic metrics via an automated, validated cone identification algorithm. Furthermore, we were able to demonstrate high intersession repeatability of automated cone analysis on HMM™ images acquired in ART™ mode. Eventually, the HMM™ ART™ setting appeared to help visualizing cones in the presence of retinal pathology.

Main limitations of our study are the inability of Spectralis™ HMM™ to resolve cones in the foveal center, our small sample size, and the absence of reliability testing of HMM™ imaging in the presence of pathology by AO. Reliability of HMM™ imaging in diseased retinas would undoubtedly be best assessed by comparing data on HMM™ to data taken with AO-SLO of the same retinal area. Unfortunately, an AO device was not available for our study. Therefore, we chose to first test the reliability of our software on an open access AO dataset and applied spectral domain OCT to compare the situation of the retinal photoreceptor layers to the appearance on HMM™ images. However, to allow for HMM™-based photoreceptor metrics to be used in clinical trials, a normative database of HMM™ metrics needs to be established, based on a large healthy cohort, divided into age and refraction-dependent groups [9]. The results of our proof-of-concept study may provide as a first indication that the development of such a normative database is feasible.

**Fig. 5:**
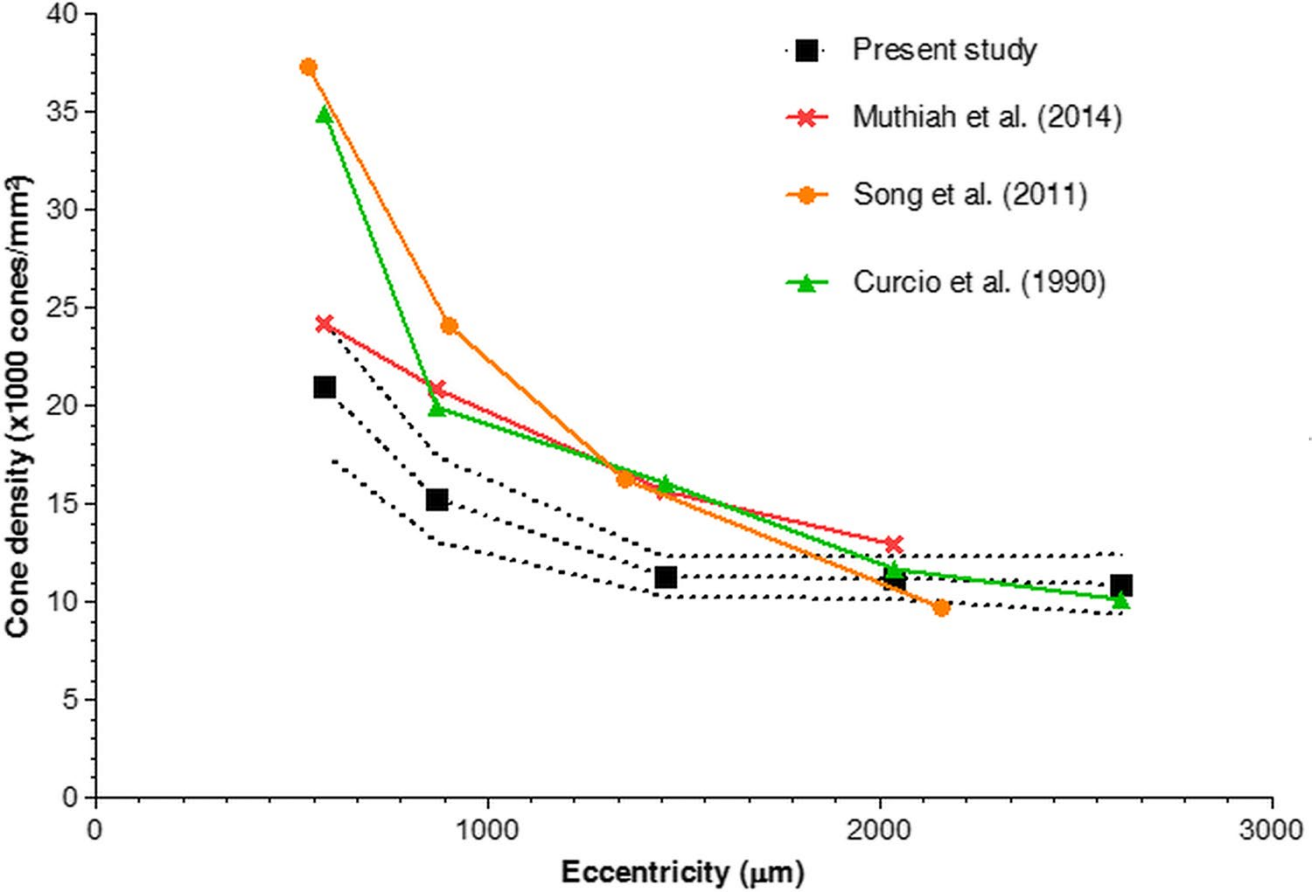
Mean temporal cone density measurements on HMM™ (our present study), AO (Muthiah et al. 2014; Song et al. 2011), and histology (Curcio et al. 1990) [23–25]. The upper and lower segmented lines represent the 95% CI upper and lower limits of our present study, respectively

Figure 5 displays a comparison of the mean temporal cone density at various eccentricities, measured by HMM™, AO, and histological studies [22–24]. The resolution of Spectralis™ HMM™ implies that only cones located in the parafovea and periphery can be resolved. In contrast, AO-based imaging modalities with a higher resolution are capable of capturing images of cones located closer to the fovea [23]. Even though, HMM™ cone density measurements between 2 and 5° are in absolute terms lower than AO and histological data, our topographical data resemble the rate of changes of HMM™ density measurements and corresponding trendlines of Curcio et al. and Song et al. Both studies applied considerably smaller ROIs for the measurements compared to Muthiah et al. and to our present study, indicating that cone numbers in the near vicinity of the fovea may be measured more precisely compared to more peripheral retinal locations if smaller ROIs are used. Therefore, one may wish to adapt ROI size for cone analysis dependent on their eccentricity on HMM™ images to avoid underestimation of cones.

NND analysis revealed a range of 5.30–8.51 μm between 5 and 9°. This closely resembles the histological findings by Curcio et al., who reported 6–8 μm at eccentricities beyond 1 mm in a 35-year-old male corneal transplant donor [25]. These results indicate that metrics evaluating structural arrangements of cone mosaic may also be reliably assessed on HMM™ imaging.

Our automatically revealed cone density measurements are similar to the results of Mendonça et al., who manually counted photoreceptors on HMM™ images [9]. However, we were able to demonstrate a considerably better intersession repeatability of cone metrics, which may be the result of a more reliable positioning of ROIs related to the fovea in our present study. We defined the foveal center by OCT cube overlay; however, Mendonça et al. only used the HMM™ images for this purpose [9]. Here, it is important to note that we have observed significant topographical differences between the foveal center on OCT and the presumed foveal center on HMM™ images. Therefore, we think that HMM™ images alone are not suitable enough to define the location of the foveal center for further analysis.

Performance of our automated cone identification methodology was compared to manual cone counts from a previous AO study in healthy probands [20]. No statically significant difference in cone density count was found. However, we observed under sampling of cones by our algorithm in the presence of pathology on HMM™ images. It is possible that cones of lower reflectivity may be missed by our algorithm that is based upon detection of maxima. Further refinement of our algorithm or, alternatively, manual count may be required in pathology. However, in our opinion, computer-assisted automated measurements should be preferred in HMM™ analysis due to their independence of human grader variability and the possibility to analyze large datasets [26].

In our current study, we found that automated computer-assisted cone analysis on HMM™ images is possible in a reliable and repeatable manner, which could not earlier been demonstrated with this imaging technique. Photoreceptor metrics based on the cone mosaic could be derived from HMM™ images and photoreceptors were repeatedly visible in retinal diseases. Even though HMM™ imaging will not be able to completely replace AO imaging due to its lower resolution and, thus, inability to investigate foveal cones, because HMM™ imaging is straightforward and relative inexpensive, it appears beneficial to detect retinal disease progression markers on a cellular level in upcoming studies alongside AO-based systems.

## Data Availability

The datasets analyzed during the current study are available from the corresponding author on reasonable request.

## References

[1] Liang J, Williams DR, Miller DT (1997). Supernormal vision and high-resolution retinal imaging through adaptive optics. J Opt Soc Am A.

[2] Liang J, Grimm B, Goelz S, Bille JF (1994). Objective measurement of wave aberrations of the human eye with the use of a Hartmann-Shack wave-front sensor. Opt Soc Am A Opt Image Sci Vis.

[3] Burns SA, Elsner AE, Sapoznik KA, Warner RL, Gast TJ (2019). Adaptive optics imaging of the human retina. Prog Retin Eye Res.

[4] Marcos S, Werner JS, Burns SA, Merigan WH, Artal P, Atchison DA (2017). Vision science and adaptive optics, the state of the field. Vision Res.

[5] Litts KM, Cooper RF, Duncan JL, Carroll J (2017). Photoreceptor-based biomarkers in AOSLO retinal imaging. Invest Ophthalmol Vis Sci.

[6] LaRocca F, Dhalla AH, Kelly MP, Farsiu S, Izatt JA (2013). Optimization of confocal scanning laser ophthalmoscope design. J Biomed Opt.

[7] Jayabalan GS, Kessler R, Fischer J, Bille JF, Bille JF (2019). Compact adaptive optics scanning laser ophthalmoscope with phase plates. High resolution imaging in microscopy and ophthalmoscopy.

[8] Vasseur V, Arej N, Alonso AS, Lafolie J, Philibert M, Vignal-Clermont C (2020). Spectralis high magnification module imaging in a case of multiple evanescent white dot syndrome. Am J Ophthalmol Case Rep.

[9] Mendonça LSM, Braun PX, Martin SM, Hüther A, Mehta N, Zhao Y (2020). Repeatability and reproducibility of photoreceptor density measurement in the macula using the Spectralis High Magnification Module. Ophthalmol Retina.

[10] Konstantinou EK, Mendonça LSM, Braun P, Monahan KM, Mehta N, Gendelman I (2020). Retinal imaging using a confocal scanning laser ophthalmoscope-based high magnification module. Ophthalmol Retina.

[11] Schindelin J, Arganda-Carreras I, Frise E, Kaynig V, Longair M, Pietzsch T (2012). Fiji: an open-source platform for biological-image analysis. Nat Methods.

[12] Preibisch S, Saalfeld S, Tomancak P (2009). Globally optimal stitching of tiled 3D microscopic image acquisitions. Bioinformatics.

[13] Bogovic JA, Hanslovsky P, Wong A, Saalfeld S (2016) Robust registration of calcium images by learned contrast synthesis. ISBI. Prague, Czech Republic. IEEE: 1123–1126. 10.1109/ISBI.2016.7493463

[14] Hirsch J, Curcio CA (1989). The spatial resolution capacity of human foveal retina. Vision Res.

[15] Ollion J, Cochennec J, Loll F, Escudé C, Boudier T (2013). TANGO: a generic tool for high-throughput 3D image analysis for studying nuclear organization. Bioinformatics.

[16] Scoles D, Sulai YN, Langlo CS, Fishman GA, Curcio CA, Carroll J, Dubra A (2014). In vivo imaging of human cone photoreceptor inner segments. Invest Ophthalmol Vis Sci.

[17] Cooper RF, Wilk MA, Tarima S, Carroll J (2016). Evaluating descriptive metrics of the human cone mosaic. Invest Ophthalmol Vis Sci.

[18] Brocher J (2015). The BioVoxxel image processing and analysis toolbox.

[19] Cunefare D, Fang L, Cooper RF, Dubra A, Carrol J, Farsiu S (2017). Open source software for automatic detection of cone photoreceptors in adaptive optics ophthalmoscopy using convolutional neural networks. Sci Rep.

[20] Garrioch R, Langlo C, Dubis AM, Cooper RF, Dubra A, Carroll J (2012). Repeatability of in vivo parafoveal cone density and spacing measurements. Optom Vis Sci.

[21] Carroll J, Baraas RC, Wagner-Schuman M, Rha J, Siebe CA, Sloan C (2009). Cone photoreceptor mosaic disruption associated with Cys203Arg mutation in the M-cone opsin. Proc Natl Acad Sci.

[22] Curcio CA, Sloan K, Kalina RE, Hendrickson AE (1990). Human photoreceptor topography. J Comp Neurol.

[23] Song H, Chui TYP, Zhong Z, Elsner AE, Burns SA (2011). Variation of cone photoreceptor packing density with retinal eccentricity and age. Invest Ophthalmol Vis Sci.

[24] Muthiah MN, Gias C, Chen FK, Zhong J, McClelland Z, Sallo FB (2014). Cone photoreceptor definition on adaptive optics retinal imaging. Br J Ophthalmol.

[25] Curcio CA, Sloan KR (1992). Packing geometry of human cone photoreceptors: variation with eccentricity and evidence for local anisotropy. Vis Neurosci.

[26] van Grinsven MJ, Lechanteur YT, van de Ven JP, van Ginneken B, Hoyng CB, Theelen T (2013). Automatic drusen quantification and risk assessment of age-related macular degeneration on color fundus images. Invest Ophthalmol Vis Sci.

